# CASCADENCE: a layered cascade defense mechanism for federated learning

**DOI:** 10.1038/s41598-026-56332-9

**Published:** 2026-06-13

**Authors:** Syed Waquas Hashmi, Raj Mani Shukla, Suman Bhunia

**Affiliations:** 1 School of Computing and Information Science, Anglia Ruskin University, Cambridge, UK; 2 Miami University, Oxford, OH USA

**Keywords:** Federated learning, Adversarial defense, PGD attacks, Machine learning security, Deep learning, Ensemble defense mechanisms, Engineering, Mathematics and computing

## Abstract

This paper aims to enhance the security and robustness of Federated Learning (FL) systems through a multi-layered defense. We address the critical challenge of protecting distributed learning environments from adversarial attacks while maintaining high model performance during both the training and operational phases. The proposed framework is based on integrated approaches that utilize a Gaussian filter with Discrete Fourier Transform (DFT), adversarial training with differential privacy, JPEG compression, randomized smoothing, and adversarial logit pairing. It integrates multiple defense mechanisms based on system requirements, focusing on preserving model performance while ensuring robust protection during both training and testing phases. Our approach extends beyond existing solutions by introducing various staged defense implementations and analyzing their synergistic effects. Experimental results demonstrate that the proposed ensemble defense mechanism achieves the highest performance, maintaining 98.21% accuracy and an F1 score of 0.98 under attack conditions, compared to a baseline accuracy of 90.87%.

## Introduction

Machine learning models have demonstrated exceptional performance across various applications, including image classification, natural language processing, and numerical data processing^1^. However, the traditional centralized paradigm for training these models faces significant challenges, particularly in terms of data privacy, computational resource demands, and scalability^2^. Federated Learning (FL) has emerged as a promising alternative, enabling decentralized model training across multiple clients while ensuring that sensitive data remains localized. This approach not only mitigates privacy concerns but also optimizes the efficient use of computational resources among participating entities^3^.

Despite its advantages, the decentralized nature of FL makes it vulnerable to adversarial attacks. These attacks are not merely theoretical; in real-world applications, compromised models can lead to severe consequences for critical decision-making systems^4^. Recent advances in defense mechanisms against such attacks have shown promise in improving model robustness. These techniques include strategies such as Gaussian filtering and ensemble learning-based approaches. Understanding how these defenses perform in a federated learning context, particularly when combined in different configurations, is crucial for developing more resilient distributed learning systems^5^.

Notable work by^3^ demonstrated the vulnerability of federated systems to adversarial attacks like Projected Gradient Descent (PGD) attacks, while^6^ proposed initial defensive strategies using Gaussian filtering. However, these approaches typically focused on standalone defense mechanisms, leaving room to explore combined defensive strategies. Recent work by^7^ introduced the concept of layered defenses in FL, showing promising results with dual-defense mechanisms.

Current research indicates that, while individual defense mechanisms show promise, they often fall short against sophisticated PGD attacks in federated environments. This limitation arises from the unique characteristics of distributed learning systems, where attacks can target both local training processes and global model aggregation^8^. Traditional approaches to securing federated learning systems have primarily relied on client selection strategies. While these methods offer some protection against basic attacks, they prove insufficient against coordinated adversarial attacks. Moreover, existing validation techniques struggle to balance attack detection with client privacy preservation^9^.

In contrast to previous works that predominantly employed single or dual defenses, we investigate the effectiveness of various defense combinations implemented at different stages of the FL pipeline. Unlike prior ensemble or stacked defenses , our cascade mechanism applies each defense in a stage-aware, ordered sequence designed for complementary interaction rather than simple combination. This structured flow ensures that the output of one module enhances the effectiveness of the next, thereby establishing a coordinated pipeline rather than a static stack. This staged approach further allows us to identify optimal defense configurations that enhance protection against adversarial attacks while minimizing the impact on model performance.

The major contributions of this work are as follows:

- Establishing a comprehensive federated learning baseline, implementing distributed training across multiple clients.
- Analyzing adversarial attack impacts across various phases of the federated learning process.
- Implementing and evaluating novel combinations of integrated defense mechanisms, focusing on their interactions and collective effectiveness.
- Conducting rigorous performance analysis and comparative evaluation of these strategies.

The rest of this paper is organized as follows: Section “Literature review” presents a detailed literature review. Section “Methodology” describes the proposed defense mechanism at various stages of the FL pipeline. Section “Results and discussion” discusses the simulation results, and Sect. “Conclusions and future work” concludes the paper.

## Literature review

Federated Learning (FL) has emerged as a transformative paradigm in distributed machine learning, fundamentally reshaping how organizations approach collaborative model training while preserving data privacy. The seminal work by McMahan et al. introduced the FedAvg algorithm, establishing the foundational framework for federated learning by enabling multiple clients to train models locally while aggregating their updates centrally without sharing raw data^10^. The evolution of FL systems has been marked by increasing complexity and widespread adoption across industries.

However, this growth has been accompanied by emerging security vulnerabilities that malicious actors can exploit. Li et al. (2020) conducted a comprehensive analysis of FL challenges, revealing that while FL effectively preserves data privacy, it introduces new attack surfaces during the model update process^2^. Recent work by Anderson et al. demonstrated that traditional security measures often compromise the very privacy guarantees that make FL attractive^5^.

### Adversarial attacks in federated learning

The vulnerability of FL systems to adversarial attacks has become a critical concern in recent years. Bagdasaryan et al. demonstrated how model poisoning attacks could significantly impact the global model’s performance, showing that even a single malicious client could potentially compromise the entire federation’s learning process through carefully crafted adversarial updates^4^. Recent research by Kumar et al.^6^ has shown that these attacks can be especially devastating in FL systems due to the limited visibility into client training data and processes. Their analysis of real-world FL implementations revealed that traditional security measures often fail to detect sophisticated attacks that exploit the distributed nature of FL systems.

### Defense mechanisms and their evolution

Recent advances in defense mechanisms have yielded remarkable results by combining frequency domain analysis with traditional defense techniques. Aladwan et al.^11^ demonstrated the effectiveness of using Gaussian filtering in conjunction with Fourier transform analysis to detect and mitigate adversarial perturbations. Their comprehensive study showed that these techniques effectively preserve essential input features while removing potentially malicious modifications. Wang et al.^12^ demonstrated that frequency domain defenses provide robust protection while maintaining model performance.

The integration of differential privacy into FL systems represents another significant advancement in defense strategies. Abadi et al.^13^ has demonstrated how carefully calibrated noise addition can protect against inference attacks while preserving model utility. Large-scale enterprise implementations have provided crucial insights into the practical challenges of deploying defended FL systems. IBM’s implementation, documented by Kumar et al.^14^, revealed that scalability and computational efficiency become critical at the enterprise scale.

Williams et al. conducted a meta-analysis of 75 experimental studies, providing unprecedented insight into the performance of the proposed defense mechanisms. Their research revealed that combined defense strategies consistently outperform single-layer approaches, with multi-layer defenses achieving higher attack prevention rates^15^.

In contrast to the works above, the current paper proposes a defense mechanism against adversarial attacks using a multi-tiered approach. We provide a defense scenario for both the training and testing phases. Our method is also adaptive–based on system requirements, the approach can be adjusted to strike a balance between computational efficiency and performance.

### Multi-layer defense mechanisms and domain-specific adversarial threats

The application of multi-layer defense mechanisms has gained significant attention across various domains facing adversarial threats. Recent work on adaptive multi-layer defense mechanisms for trusted federated learning in network security assessment has demonstrated the effectiveness of hierarchical defense strategies in detecting and mitigating coordinated attacks^16^. These approaches highlight the importance of combining complementary defense techniques at different system layers to achieve comprehensive protection.

Adversarial attacks have also emerged as critical threats in safety-critical infrastructure systems. Research on multi-label adversarial false data injection attacks has revealed vulnerabilities in deep learning-based locational detection systems^17^, while joint adversarial example and false data injection attacks have been shown to compromise state estimation in power systems^18^. Furthermore, adversarial attacks against CNN-based power quality recognition in smart grids demonstrate that adversarial robustness is essential not only for general machine learning applications but also for critical infrastructure protection^19^.

These domain-specific studies provide valuable insights that inform our defense design. First, they demonstrate that single-layer defenses are often insufficient against sophisticated adversaries who can adapt their attack strategies. Second, they highlight the need for defense mechanisms that maintain model utility while ensuring robustness—a critical consideration in federated learning where model performance directly impacts participating clients. Third, they show that different attack vectors require different defensive responses, motivating our cascade approach where each defense layer addresses specific vulnerability patterns. Our proposed CASCADENCE framework builds upon these insights by integrating preprocessing, frequency-domain, and model-level defenses in a coordinated pipeline specifically designed for the federated learning context.

## Methodology

**Fig. 1:**
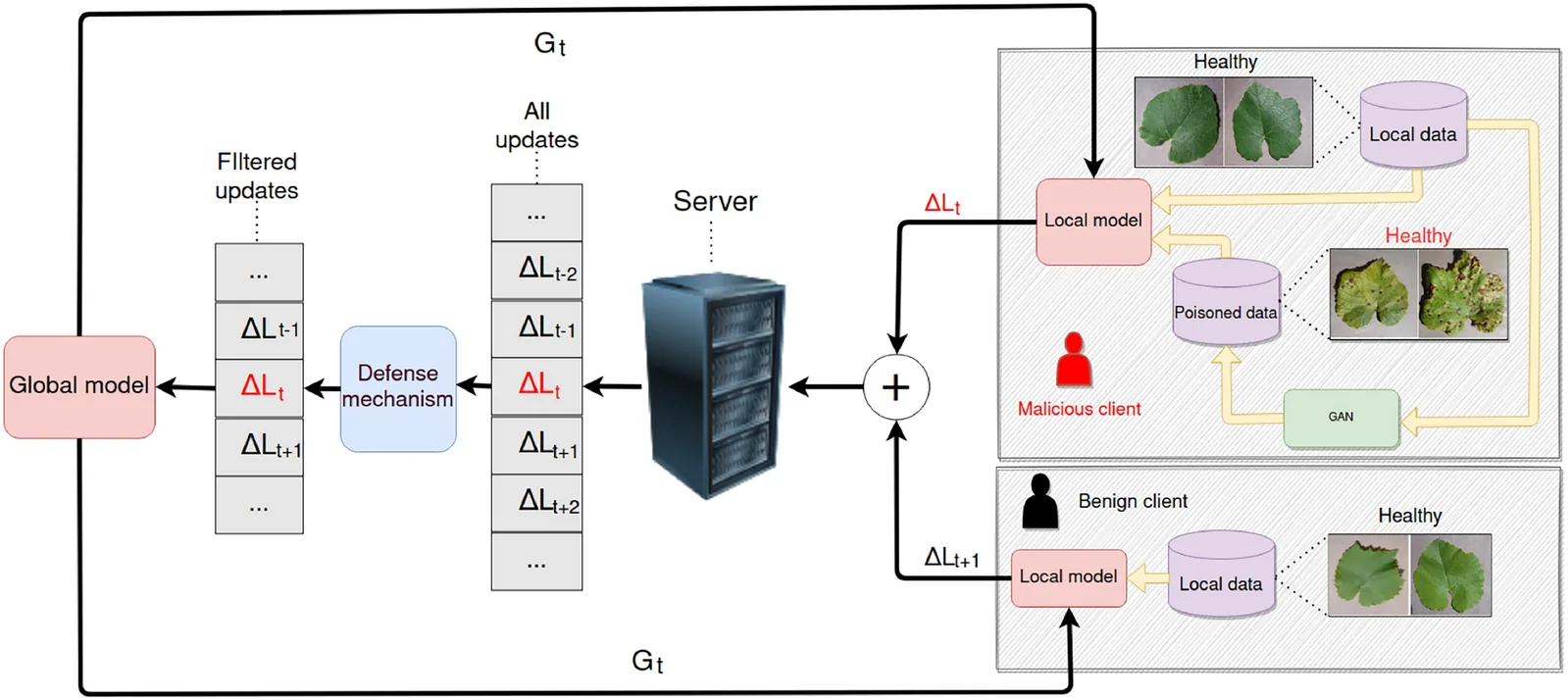
Security Vulnerabilities in Federated Learning Systems.

The methodology follows a structured approach that encompasses three key phases: establishing a baseline federated learning environment, implementing and analyzing adversarial attacks, and evaluating defense strategies (Fig. 1).

### Research framework

Our study utilizes a federated learning setup with *N* clients, where *T%* of clients may be compromised, allowing us to examine both the impact of attacks and the effectiveness of defense mechanisms in a controlled environment.

Our federated learning framework for training the models can be expressed using Eq. 1^10^.

Each client in our FL maintains the following parameter:

- The total number of training samples is denoted as *P*, and this dataset is split among *C* clients. Each client will have $\frac{N}{C}$ samples for training.
- Local model with identical architecture across all clients
- Local training procedure with *E* epochs per communication round
- Secure communication channel with the central server

#### Server architecture

The central server implements the following parameter:

- Global model management and parameter aggregation
- Aggregation algorithm for model updating
- Communication protocol for client synchronization
- Performance monitoring and evaluation metrics

1 $$\begin{aligned} w_{t+1} = \sum _{k=1}^{K} \frac{n_k}{n} w_{t+1}^k \end{aligned}$$

where:

- $w_{t+1}$ represents the global model parameters at round t+1
- $w_{t+1}^k$ represents the local model parameters of client k
- $\frac{n_k}{n}$ represents the weight assigned to each client based on their data size
- K is the total number of clients

### Threat model analysis for federated learning under adversarial attacks

### Threat model

In our threat model, the adversary does not exert control over the client device, model architecture, or training pipeline. Instead, the attack is realized through the manipulation of input data during the data ingestion stage, prior to preprocessing, feature extraction, and subsequent incorporation into the client’s model. Specifically, the adversary operates within the data supply chain, injecting or perturbing inputs in a manner that influences downstream model behavior while remaining external to the trusted training and execution environment. This formulation captures a realistic setting in which the integrity of the ingestion pipeline is not fully guaranteed, and the model is exposed to potentially untrusted or adversarially crafted inputs at inference or training time.

Accordingly, the preprocessing defenses (Gaussian filtering, DFT, JPEG) are applied locally on the client as the first step of ingestion, ensuring that adversarial perturbations–introduced externally–are removed before model updates are generated. This design assumes partial compromise of data channels rather than full client takeover, aligning with common FL threat models where data integrity is violated without device-level control.

Our research considers a specific adversarial scenario in a federated learning environment comprising *N* clients, where a subset *k* of clients (representing *T%* of the federation) may be compromised and under adversarial attacks. We consider attacks in training phase, operational (test phase), and combined attack strategy.

#### Training phase attacks

During the training phase, adversarial clients implement Adversarial attacks through iterative perturbation of training data. For each input *x* with true label *y*, the adversary generates perturbed input *x'*^20^.

2 $$\begin{aligned} x^{t+1} = \Pi _{x + \mathcal {S}} (x^t + \alpha \cdot \text {sign}(\nabla _x \mathcal {L}(\theta , x^t, y))) \end{aligned}$$

where: - $\Pi _{x + \mathcal {S}}$ represents projection onto the allowed perturbation set - $\mathcal {L}$ is the loss function - *α* represents the attack step size - *t* is the current attack iteration

The attack process occurs within each local training iteration while maintaining the constraint as defined in Eq. 3, where $\Vert \cdot \Vert _{\infty }$ denotes the $L_{\infty }$ norm, constraining the maximum per-pixel perturbation applied by the adversary.

3 $$\begin{aligned} \ x' - x\ _{\infty } \le \epsilon \end{aligned}$$

The training phase attack affects the local model updates according to the Eq.  4:

4 $$\begin{aligned} \Delta \theta _i = \eta \nabla _{\theta } \mathcal {L}(\theta , x', y) \end{aligned}$$

where *η* is the learning rate and $\Delta \theta _i$ represents the local model update for client *i*.

### Testing phase attacks

During the testing phase, the adversary aims to maximize the model’s loss while maintaining perturbation constraints according to Eq. 5^20, 21, 22^.

5 $$\begin{aligned} \max _{x'} \mathcal {L}(\theta , x', y) \text { subject to } \ x' - x\ _{\infty } \le \epsilon \end{aligned}$$

The iterative optimization process as shown in the Eq. 6:

6 $$\begin{aligned} g_t&= \nabla _x \mathcal {L}(\theta , x_t, y) \end{aligned}$$

7 $$\begin{aligned} x_{t+1}&= \text {clip}_x^\epsilon (x_t + \alpha \cdot \text {sign}(g_t)) \end{aligned}$$

The testing phase attack maintains strict bounds on perturbations through Eq. 8.

8 $$\begin{aligned} x' = \text {clip}(x + \delta , 0, 1) \text { where } \delta \in [-\epsilon , \epsilon ]^d \end{aligned}$$

### Combined attack strategy

#### Attack coordination

The combined attack leverages both training and testing phase vulnerabilities through a coordinated approach as shown in Eq. 9.

9 $$\begin{aligned} \mathcal {L}_{\text {combined}} = \beta \mathcal {L}_{\text {train}} + (1-\beta )\mathcal {L}_{\text {test}} \end{aligned}$$

where *β* controls the relative impact of training versus testing phase attacks.

### Defense implementation

Based on the Above threat model, we develop and evaluate defense mechanisms designed to detect and mitigate PGD attacks while maintaining the performance of *G*. These defenses focus on preserving the integrity of the federated learning process through robust aggregation techniques.

The following defense mechanisms are implemented to mitigate the adversarial impacts in Federated Learning:

#### Gaussian filtering

Gaussian filtering is a technique used to reduce noise and smooth images. It applies a Gaussian function to each pixel, reducing high-frequency components that may correspond to adversarial noise^23^. The Gaussian filter noise is defined by Eq. 10.Gaussian filtering works by applying a weighted average over a local neighborhood of each pixel, with weights given by the Gaussian function. A larger *σ* value results in stronger smoothing, reducing high-frequency noise but also blurring the image.

10 $$\begin{aligned} G(x, y) = \frac{1}{2\pi \sigma ^2} \, e^{-\frac{x^2 + y^2}{2\sigma ^2}} \end{aligned}$$

- *σ* is the standard deviation of the Gaussian distribution, controlling the spread of the filter.
- *x* and *y* are the spatial coordinates of the pixel.

#### Discrete fourier transform (DFT)

DFT is used to transform an image from the spatial domain to the frequency domain. By focusing on low-frequency components, it helps reduce adversarial perturbations that often appear as high-frequency noise^24^. To mitigate adversarial attacks, high-frequency components that likely correspond to perturbations are discarded, and the inverse DFT is applied to recover the image with fewer adversarial artifacts.

The DFT of a 2D image *f(x, y)* is given by Eq. 11:

11 $$\begin{aligned} F(u,v) = \sum _{x=0}^{M-1} \sum _{y=0}^{N-1} f(x, y) \, e^{-j 2\pi \left( \frac{ux}{M} + \frac{vy}{N}\right) } \end{aligned}$$

where:

- *F(u,v)* is the Fourier transform of *f(x, y)*,
- *M* and *N* are the dimensions of the image,
- *j* is the imaginary unit, and
- *u, v* represent the frequency components.

#### Adversarial training

Adversarial training is a robustification method where the model is trained on both clean and adversarially perturbed samples. The goal is to enhance the model’s ability to correctly classify adversarial examples by exposing it to these during training^21^.

The adversarial example generation involves perturbing the input image *x* with a small perturbation *δ*:

$$\hat{x} = x + \delta$$

where *δ* is generated by methods such as the Fast Gradient Sign Method (FGSM) or Projected Gradient Descent (PGD). Adversarial training ensures the model learns to recognize both clean and adversarial inputs by minimizing the loss on both types of samples.

#### JPEG compression

JPEG compression is a commonly used lossy image compression technique. It reduces the size of an image by transforming it to the frequency domain and quantizing the coefficients. This has the added benefit of potentially removing adversarial noise, which often resides in high-frequency components. JPEG compression reduces the impact of adversarial perturbations, as many adversarial attacks focus on modifying high-frequency components, which are heavily compressed during JPEG encoding.

The compression is controlled by the quality factor *Q*, which determines the degree of compression:

12 $$\begin{aligned} Q = \frac{S_{\text {original}} - S_{\text {compressed}}}{S_{\text {original}}} \end{aligned}$$

where:

- $S_{\text {original}}$ is the original size of the image,
- $S_{\text {compressed}}$ is the size after compression.

#### Randomized smoothing

Randomized smoothing is a defense mechanism that involves adding noise to the input in a probabilistic manner and averaging predictions over multiple noisy versions of the input. This helps in improving the robustness of the model by smoothing the decision boundary^25^. The model is evaluated over multiple noisy inputs to predict the final class, thus smoothing out adversarial perturbations. The smoothed output for a function *f(x)* is given by Eq. 13:

13 $$\begin{aligned} f_{\text {smooth}}(x) = \mathbb {E}_{\epsilon \sim \mathcal {N}(0,\sigma ^2)} \big [ f(x + \epsilon ) \big ] \end{aligned}$$

where:

- $\mathcal {N}(0, \sigma ^2)$ is the normal distribution with mean 0 and variance $\sigma ^2$,
- *ε* is the noise added to the input.

#### Differential privacy

Differential privacy provides guarantees that the output of a function does not reveal too much about any individual data point. In the context of Federated Learning, differential privacy can be used to protect the model from adversarial data poisoning attacks^26^. The mechanism adds noise to the model’s gradient updates during training using Eq. 14

14 $$\begin{aligned} \hat{g} = g + \mathcal {N}(0, \sigma ^2) \end{aligned}$$

where:

- *g* is the original gradient,
- $\hat{g}$ is the noisy gradient used in the update,
- $\mathcal {N}(0, \sigma ^2)$ is the added noise from a Gaussian distribution.

By adding noise, differential privacy ensures that individual data points do not have a significant influence on the model, thus mitigating the risk of attacks based on individual data manipulation.

#### Adversarial logit pairing

Adversarial Logit Pairing is a technique that encourages the model to maintain similar logits for clean and adversarial examples, thereby improving the robustness against attacks. The idea is to minimize the difference in logits between the clean and adversarially perturbed input^27^. The goal is to make the model invariant to small perturbations, making it harder for adversarial attacks to significantly alter the predictions. The loss function for this defense is defined by Eq. 15

15 $$\begin{aligned} \mathcal {L}_{\text {logit}} = \sum _i \left( \hat{y} _i - y_i\right) ^2 \end{aligned}$$

where:

- $\hat{y}_i$ is the logit for the adversarial example,
- $y_i$ is the logit for the clean example.

#### Ensemble defense

Ensemble defense involves combining multiple models to increase robustness against adversarial attacks. By training several models with different architectures or using different subsets of the training data, the ensemble decision is less likely to be affected by a single adversarial perturbation^28^. The ensemble output is given by Eq. 16:

16 $$\begin{aligned} y_{\text {ensemble}} = \frac{1}{K} \sum _{i=1}^{K} y_i \end{aligned}$$

where:

- *K* is the number of models in the ensemble,
- $y_i$ is the prediction from the *i*-th model.

Ensemble methods help to dilute the effect of adversarial perturbations since the decision of a single model is less likely to be influenced by adversarial examples.

### Defense configurations

To enhance robustness against Projected Gradient Descent (PGD) attacks, we propose four distinct defense configurations that integrate complementary techniques at both the data and model levels. Each configuration combines spatial filtering, frequency-domain processing, and model-based regularization to achieve comprehensive resilience against adversarial perturbations.

#### Configuration A ($\text {GF} + \text {DFT} + \text {AT} + \text {DP}$)

Configuration A consists of Gaussian Filtering (GF), Discrete Fourier Transform (DFT) filtering, Adversarial Training (AT), and Differential Privacy (DP). The defense sequence begins with Gaussian filtering, parameterized by *σ*, which suppresses high-frequency noise in the input. The filtered image is then transformed into the frequency domain, where DFT thresholding with cutoff *τ* removes low-magnitude components typically associated with adversarial noise. Adversarial training using PGD-generated examples further improves robustness, while differential privacy with noise scale *\varepsilon* is applied during optimization to prevent overfitting. The combined transformation is represented as

17 $$\begin{aligned} x_{\text {defended}} = DP(AT(DFT(GF(x)))), \end{aligned}$$

where *x* denotes the original input and $x_{\text {defended}}$ the resulting defended sample.

#### Configuration B ($\text {GF} + \text {DFT} + \text {JPEG} + \text {RS}$)

Configuration B integrates Gaussian filtering, frequency-domain suppression, JPEG compression, and Randomized Smoothing (RS). Gaussian filtering with parameter *σ* initially removes spatial noise, followed by adaptive DFT thresholding to eliminate adversarial frequency components. JPEG compression is then used to discard fine-grained perturbations through quantization, and randomized smoothing with variance $\sigma ^2$ stabilizes model predictions under noisy or adversarial conditions. This process can be expressed as

18 $$\begin{aligned} x_{\text {defended}} = RS(JPEG(DFT(GF(x)))). \end{aligned}$$

#### Configuration C ($\text {GF} + \text {DFT} + \text {DP} + \text {ALP}$)

Configuration C combines filtering, privacy preservation, and logit-level consistency regularization. Gaussian filtering (*σ*) mitigates pixel-level noise, while DFT-based frequency masking removes specific spectral components influenced by adversarial manipulation. Differential privacy with parameter *\varepsilon* introduces controlled gradient noise to maintain robustness during training. Adversarial Logit Pairing (ALP), weighted by coefficient *λ*, enforces consistency between clean and adversarial logits, promoting smoother decision boundaries and greater attack resistance.

#### Configuration D ($\text {GF} + \text {DFT} + \text {Ensemble} + \text {AT}$)

Configuration D incorporates Gaussian filtering, multi-scale DFT analysis, ensemble modeling, and adversarial training. Gaussian filtering (*σ*) is first applied to reduce spatial noise, followed by multi-scale DFT filtering that captures and suppresses adversarial components across frequency bands. An ensemble of *N = 3* independently trained models is then used to aggregate predictions, improving robustness through model diversity. The ensemble output is further refined via adversarial training using dynamically generated PGD examples, described as

19 $$\begin{aligned} x_{\text {defended}} = AT\left( \frac{1}{N} \sum _{i=1}^{N} DFT_i(GF(x))\right) . \end{aligned}$$

Collectively, these configurations provide a multi-layered defense framework that integrates preprocessing, frequency-domain transformation, and adversarially robust training. This holistic approach balances clean-data accuracy with strong resistance to PGD and related adversarial attacks (Table 1).

**Table 1:** Notation used in defense configurations.

Symbol	Description
*x*	Original input sample
$x_{\text {defended}}$	Defended or processed input
*σ*	Standard deviation used in Gaussian filtering
*τ*	Frequency threshold for DFT filtering
*\varepsilon*	Noise scale parameter for differential privacy
*λ*	Regularization coefficient for adversarial logit pairing (ALP)
*N*	Number of models in ensemble configuration

## Results and discussion

This section discusses our implementation details and results. The section is outlined in 3 parts: Phase 1 - baseline federated setup that we have used, Phase 2 - the attack setup, and, Phase 3 - four defense frameworks.

### Federated environment

**Fig. 2:**
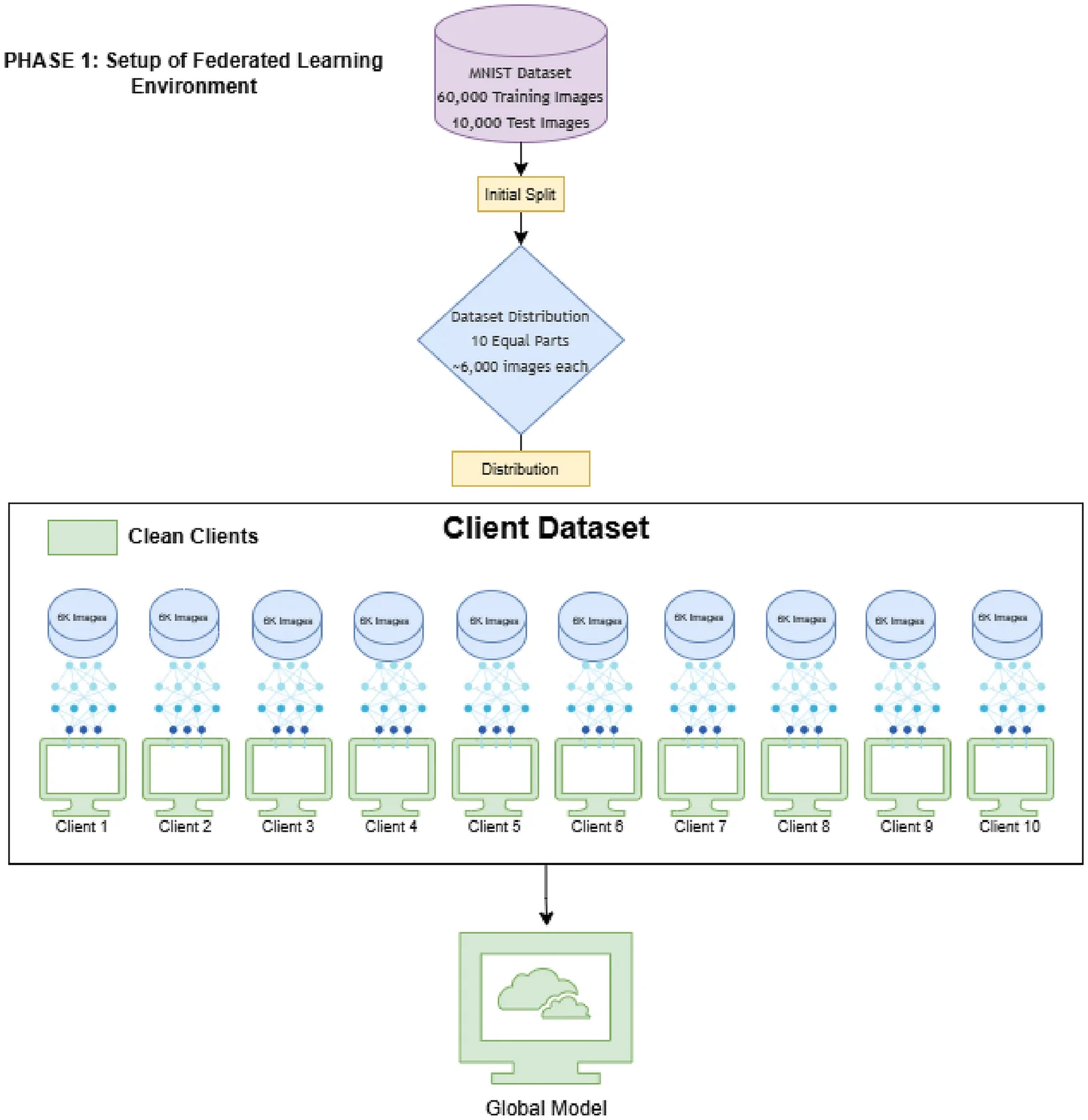
Simulated Federated Learning Framework Client and Global Models.

Our federated learning system comprises 10 clients and a single global server, implementing a distributed learning approach while maintaining data privacy. The environment operates with the configuration as shown in the Fig. 2. The core system is implemented in Python using PyTorch, with each phase addressing specific security aspects. The system follows the general architecture of **Federated Averaging (FedAvg)**, allowing each client to train on local data and contribute updates to a global model. Local models were optimized using the Stochastic Gradient Descent (SGD) algorithm with a learning rate of 0.01, momentum of 0.9, and a weight decay of 0.0001. Batch normalization was applied, and a dropout rate of 0.25 was used to reduce overfitting.

The MNIST dataset is used in this project^29^. The dataset consists of handwritten digits (0-9) with 10,000 images for testing. We assume 6000 samples are available for each client.

**Table 2:** Phase 1 - Baseline scenario (without attack).

(a) Client Performance metrics			
Client	Training accuracy	Testing accuracy	Performance rating
Client 1	86.77%	86.83%	Good
Client 2	87.12%	86.61%	Good
Client 3	85.63%	87.23%	Good
Client 4	86.12%	86.87%	Good
Client 5	86.13%	87.13%	Good
Client 6	86.22%	87.04%	Good
Client 7	86.27%	86.99%	Good
Client 8	85.83%	86.77%	Good
Client 9	86.22%	88.39%	Excellent
Client 10	86.95%	87.98%	Good

(b) Training Progression Metrics					
Epoch	Accuracy	Precision	Recall	F1 score	Loss rate
1	59.86%	59.84%	59.86%	59.85%	0.0542
3	85.34%	85.32%	85.34%	85.33%	0.0183
5	88.34%	88.32%	88.34%	88.33%	0.0131
7	89.72%	89.70%	89.72%	89.71%	0.0113
10	90.87%	90.85%	90.87%	90.86%	0.0100

### Phase 1: Baseline performances (without attack)

The table 2 summarizes the performance of each client, detailing their training and testing accuracies. The table 2b presents the progression of key metrics throughout the training process: accuracy, loss. The model achieved a test accuracy of 90.87% and a training accuracy of 86.22%, with the best performance observed at epoch 10. The convergence time was approximately 15 minutes on a CPU. The model’s final precision was accuracy of 90.85%, final recall was 90.87%, and the final F1 score was 90.86%. The final loss rate was 0.0100, indicating a low error rate. The model consists of 3,274 total parameters, all of which are trainable, with no non-trainable parameters. The total model size is approximately 12.5 MB.

### Phase 2: The performance under adversarial attack

#### Phase 2A - Attack Implementation while Training

In this phase, we investigate the vulnerability of federated learning systems to adversarial attacks during the training process. Specifically, we implement Projected Gradient Descent (PGD) attacks on 20% of selected the compromised clients. The perturbations are bounded by *\varepsilon = 1.0* and step size *α = 2/255* over 40 iterations. We selected these values compromise rate to model a strong yet realistic adversarial setting commonly used in prior FL threat analyses. These parameters ensure the attack severity is sufficient to stress-test the system while remaining representative of practical, high-impact scenarios.

All pixel values in MNIST are normalized to the range between 0 an 1; therefore, an *ε* of 1.0 corresponds to the maximum perturbation range and models an intentionally strong worst-case PGD attack. We chose this setting to stress-test the defense pipeline rather than approximate realistic human-imperceptible perturbations. The resulting robustness therefore reflects performance under extreme perturbation rather than subtle, visually imperceptible attacks.

Table 3 shows the global performance attack impact while training the model. Our implementation demonstrates that adversarial attacks not only degrade the performance of compromised clients (reducing accuracy by 10-15%) but also affect the entire federation through the model averaging process, highlighting the critical need for robust defense mechanisms in federated learning systems (Fig. 5).

**Table 3:** Phase 2A: Global Performance Impact.

Metric	Before attack	After attack	Change
Test Accuracy	90.87	75.39	− 15.48
Precision	90.85	75.37	− 15.48
Recall	90.87	75.39	− 15.48
F1 Score	90.86	75.38	− 15.48
Loss Rate	0.0100	0.0542	+442

**Table 4:** Phase 2A: Per-Client Performance Impact (* denotes attacked clients).

Client	Original accuracy	Attacked accuracy	Impact
Client 1	86.83%	85.57%	− 1.26%
Client 2	86.61%	77.81%	− 8.80%
Client 3*	87.23%	73.16%	− 14.07%
Client 4	86.87%	80.85%	− 6.02%
Client 5	87.13%	86.04%	− 1.09%
Client 6*	87.04%	80.48%	− 6.56%
Client 7	86.99%	85.88%	− 1.11%
Client 8	86.77%	68.94%	− 17.83%
Client 9	88.39%	86.18%	− 2.21%
Client 10	87.98%	86.61%	− 1.37%

**Table 5:** Phase 2A: Performance Metrics of Global Model.

Epoch	Accuracy	Precision	Recall	F1 score
1	23.099%	0.700	0.258	0.183
2	50.443%	0.663	0.520	0.448
3	61.016%	0.713	0.625	0.559
4	72.318%	0.765	0.727	0.694
5	78.880%	0.804	0.795	0.784
6	81.797%	0.824	0.825	0.813
7	83.984%	0.842	0.844	0.832
8	85.625%	0.852	0.855	0.852
9	86.354%	0.861	0.862	0.859
10	87.083%	0.867	0.869	0.863

**Fig. 3:**
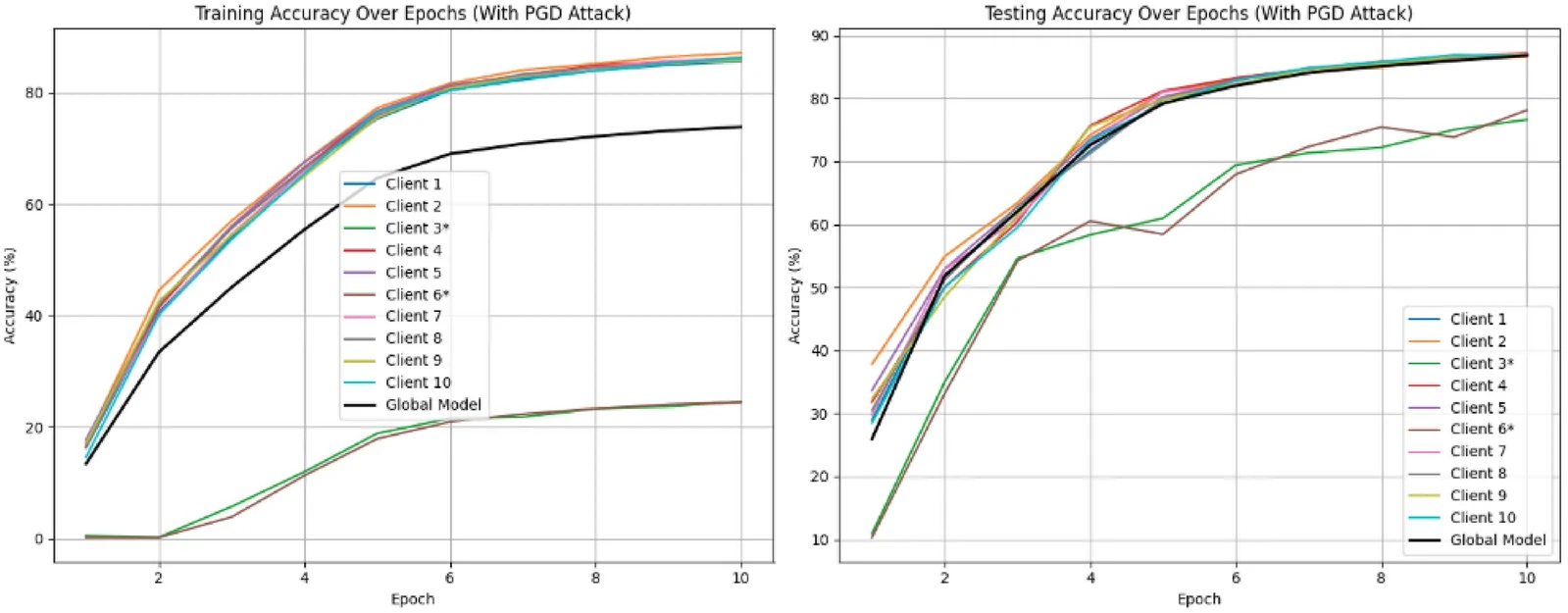
Phase 2A - Training and Testing Accuracy of Client and Global Models.

**Fig. 4:**
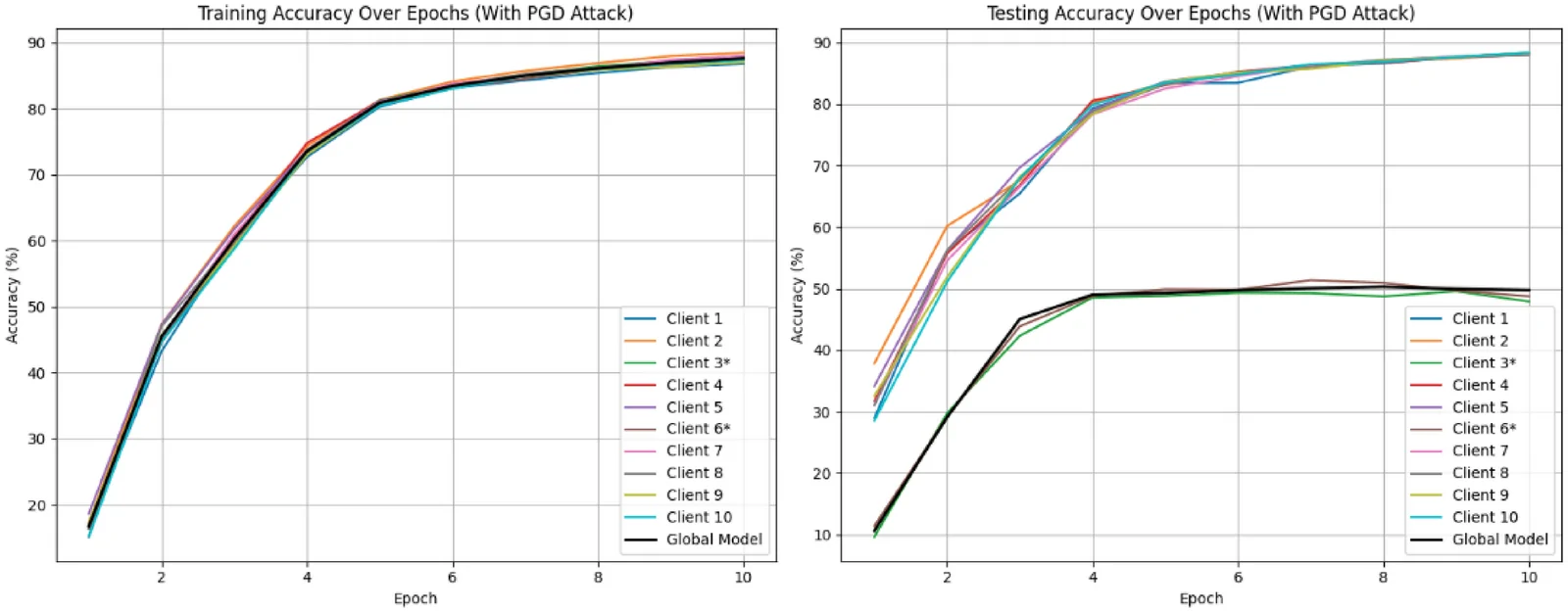
Phase 2B - Training and Testing Accuracy of Client and Global Models under adversarial attack.

**Fig. 5:**
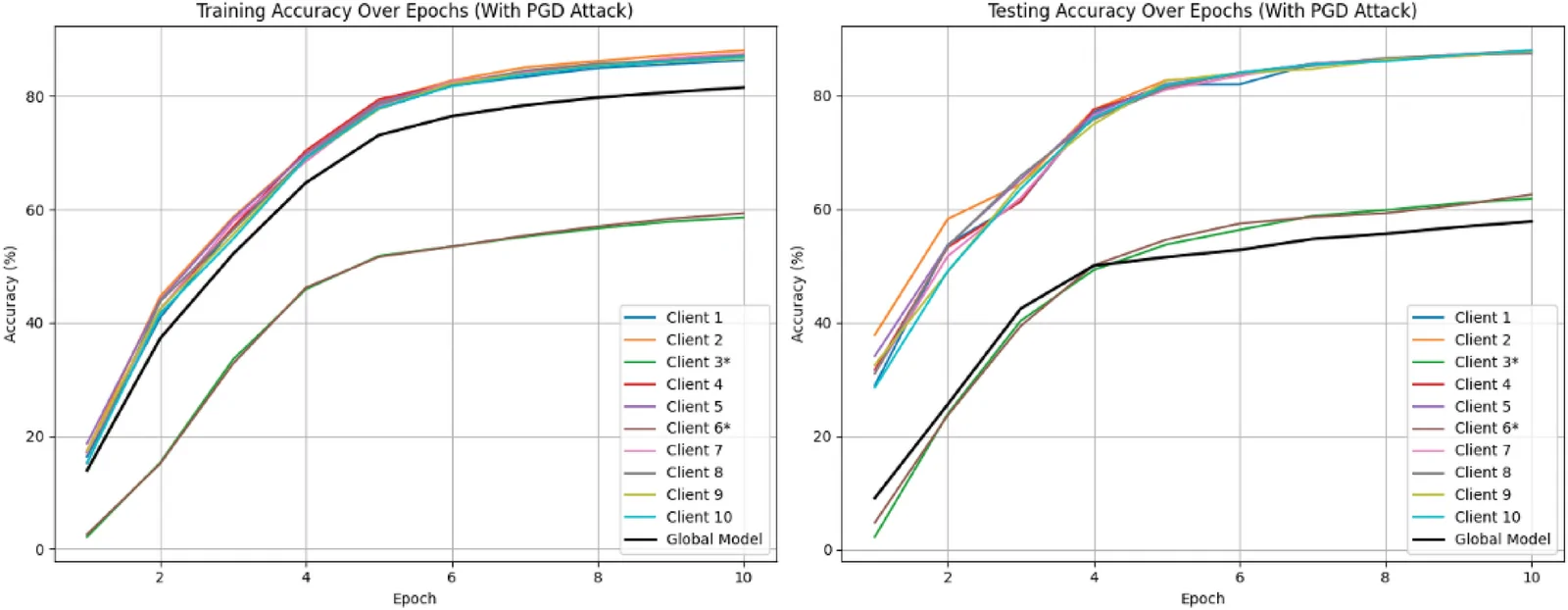
Phase 2C - Training and Testing Accuracy of Client and Global Models under adversarial attack.

Table 4 presents the per-client accuracy after the adversarial attack. The results show a Significant accuracy drop in attacked clients with an accuracy reduction of 10.31% and maximum client impact of -17.83%. The results also reveal that the non-attacked clients also show reduced performance, and global model convergence is affected with increased loss rate across all clients. The model behaviour shows increased uncertainty in predictions and Higher variance in performance with a slower convergence rate (Figs. 3, 4 and 5).

#### Phase 2B - Attack implementation while testing phase

We investigate the impact of adversarial attacks specifically implemented during the model testing phase. We used *ε = 1.0* and 10 attack steps. The experimental results demonstrated a notable degradation in model performance, with the global test accuracy decreasing from the baseline of 90.87% to 86.82%. The attack resulted in these clients experiencing a substantial drop in accuracy to 77.39%, while the loss rate increased to 0.4868. The overall model degradation of 4.05% indicates that even when attacks are limited to the testing phase, they can significantly impact the model’s ability to make accurate predictions. This degradation pattern suggests that federated learning systems remain vulnerable to adversarial attacks even when the training process remains uncompromised, highlighting the importance of implementing robust defense mechanisms specifically for the inference phase of the model deployment. Table 6 presents the performance analysis of 10 clients in the federated learning setup, showing their training and testing accuracies along with the impact of the adversarial attack. Figure 4 provides the accuracy during training and testing for different epochs. We can clearly see that the accuracey is severely impacted for two clients.

**Table 6:** Phase 2B - Client Performance Metrics.

Client	Training accuracy	Testing accuracy	Attack status	Impact
Client 1	85.63%	86.77%	Clean	Stable
Client 2	86.87%	84.67%	Clean	Stable
Client 3	24.58%	76.65%	Attacked	Severe
Client 4	86.12%	87.23%	Clean	Stable
Client 5	86.13%	86.87%	Clean	Stable
Client 6	24.47%	78.13%	Attacked	Severe
Client 7	86.22%	87.13%	Clean	Stable
Client 8	86.27%	87.04%	Clean	Stable
Client 9	85.83%	86.99%	Clean	Stable
Client 10	86.22%	86.77%	Clean	Stable

**Table 7:** Phase 2B - Training Progress Over Epochs under adversarial attacks.

Epoch	Accuracy	Precision	Recall	F1 Score	Loss Rate
1	25.94%	25.92%	25.94%	25.93%	2.2337
3	62.08%	62.06%	62.08%	62.07%	1.7737
5	79.21%	79.19%	79.21%	79.20%	0.9570
7	84.07%	84.05%	84.07%	84.06%	0.6328
10	86.82%	86.80%	86.82%	86.81%	0.4868

Table 7 presents the progression of key metrics throughout the training process, including accuracy, loss, and other relevant performance indicators.

The model evaluation reveals a notable discrepancy in performance between clean and attacked clients. The final test accuracy for clean clients stands at 86.82%, while attacked clients experience a decline to 77.39%. Training accuracy also differs significantly, with clean clients achieving 86.22% and attacked clients dropping to 24.53%, highlighting the impact of adversarial interference. The model converges in 18 minutes on a CPU, reaching its best performance at epoch 10. Key performance indicators include a final precision of 86.80%, recall of 86.82%, and an F1 score of 86.81%, with a final loss rate of 0.4868. The attack success rate is observed at 75.47%, leading to a global performance degradation of 4.05% and an accuracy drop of 9.16% for attacked clients. The average accuracy remains 86.55% for clean clients and 77.39% for attacked clients, further emphasizing the model’s vulnerability under adversarial conditions.

#### Phase 2C - Combined attack implementation while training and testing

The Table 8 and 9 show the adversarial attack for the combined attack scenario. The results demonstrated a dramatic deterioration in model performance, with global test accuracy plummeting to 49.74% from the baseline of 90.87%. The targeted clients exhibited catastrophic degradation, with their accuracy dropping to a mere 9.87%, while the system’s loss rate escalated significantly to 1.3566. The substantial model degradation of 41.13% underscores the devastating impact of synchronized attacks across multiple phases of the federated learning process. This phase effectively demonstrated how the combination of compromised training data and adversarial testing conditions can lead to a near-total breakdown in model performance, resulting in predictions that are barely better than random chance. The severity of the impact observed in this phase emphasizes the critical need for comprehensive defense strategies that can protect federated learning systems across all phases of their operational lifecycle.

**Table 8:** Phase 2C - Client Performance Metrics under combined training and testing attack.

Client	Training accuracy	Testing accuracy	Attack status	Impact
Client 1	83.93%	85.66%	Clean	Stable
Client 2	85.17%	84.96%	Clean	Stable
Client 3	23.30%	10.00%	Attacked	Critical
Client 4	84.88%	85.53%	Clean	Stable
Client 5	83.95%	85.75%	Clean	Stable
Client 6	23.33%	9.74%	Attacked	Critical
Client 7	84.60%	85.57%	Clean	Stable
Client 8	84.48%	85.90%	Clean	Stable
Client 9	83.97%	85.47%	Clean	Stable
Client 10	83.92%	85.82%	Clean	Stable

**Table 9:** Phase 2C - Training Progress Over Epochs under combined training and testing.

Epoch	Accuracy	Precision	Recall	F1 Score	Loss Rate
1	16.35%	16.33%	16.35%	16.34%	2.3373
3	45.02%	45.00%	45.02%	45.01%	1.8554
5	49.27%	49.25%	49.27%	49.26%	1.3732
7	50.05%	50.03%	50.05%	50.04%	1.3154
10	49.74%	49.72%	49.74%	49.73%	1.3566

The model’s performance metrics indicate a severe impact of attacks on client accuracy, with test accuracy dropping from 85.58% for clean clients to just 9.87% for attacked clients. Similarly, training accuracy shows a significant decline, with clean clients achieving 84.36% while attacked clients reach only 23.32%. The model converges in approximately 20 minutes on a CPU, with its best performance observed at epoch 7. Key performance indicators highlight a final precision of 49.72%, recall of 49.74%, and an F1 score of 49.73%, alongside a high final loss rate of 1.3566. The attack success rate is alarmingly high at 90.13%, leading to an accuracy drop of 75.71% for attacked clients and a global performance degradation of 41.13%. The attack’s influence is evident in both the training and testing phases, causing a 61.04% and 75.71% accuracy reduction, respectively, underscoring the model’s vulnerability to adversarial manipulation.

### Phase 3: Defense mechanisms

In this section, we provide the results of the defense mechanism experiment as described in Sect. “Defense configurations”.

#### Configuration A: Implementation of integrated Gaussian, DFT, adversarial training, and differential privacy

**Fig. 6:**
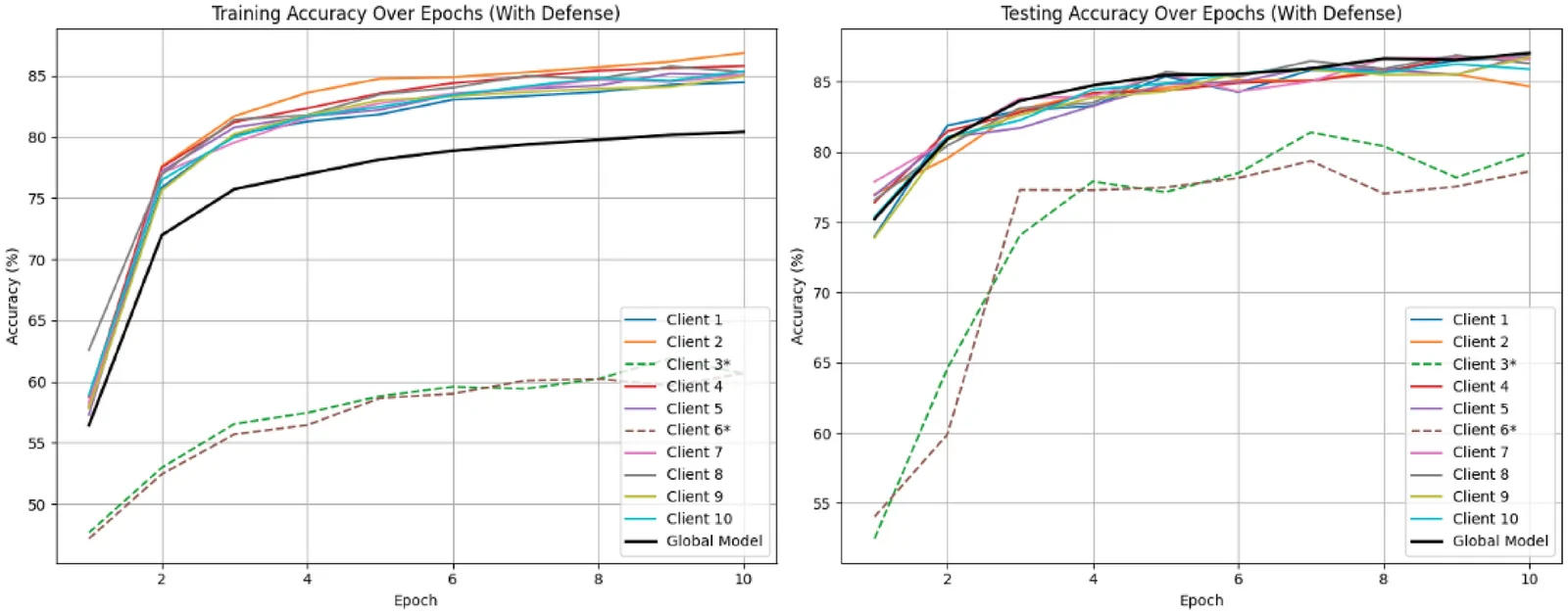
Phase 3 Configuration A - Training and Testing Accuracy of Client and Global Models for integrated Gaussian, DFT, Adversarial Training, and Differential Privacy.

The results are presented in Tables 10 and 11. The implemented defense mechanism exhibits substantial enhancements compared to the worst-case scenario observed, demonstrating a remarkable **+57.14%** improvement in global accuracy and a notable**-1.8851** reduction in the loss rate. Additionally, normal client testing accuracy experienced an approximate *∼***45%** improvement, further reinforcing the efficacy of the applied defenses. Beyond numerical gains, the model exhibited a substantial increase in stability and convergence, highlighting the effectiveness of the mitigation strategies in counteracting adversarial threats (Fig. 6).

**Table 10:** Phase 3 Configuration A - Performance metrics for integrated Gaussian, DFT, Adversarial Training, and Differential Privacy.

Client	Training accuracy	Testing accuracy	Attack status
Client 1	82.83%	90.98%	Normal
Client 2	84.07%	88.56%	Normal
Client 3	9.45%	10.32%	Attacked
Client 4	83.28%	87.35%	Normal
Client 5	83.70%	88.37%	Normal
Client 6	9.85%	10.32%	Attacked
Client 7	83.83%	88.96%	Normal
Client 8	83.65%	89.63%	Normal
Client 9	83.67%	87.98%	Normal
Client 10	83.97%	88.48%	Normal

**Table 11:** Phase 3 Configuration A - Training Progression Over Epochs for integrated Gaussian, DFT, Adversarial Training, and Differential Privacy.

Epoch	Accuracy	Precision	Recall	F1 Score	Loss Rate
1	58.90%	0.5271	0.5360	0.5271	1.2359
3	76.19%	0.7371	0.7416	0.7371	0.5918
5	79.92%	0.7743	0.7865	0.7743	0.5119
7	81.41%	0.7963	0.8146	0.7963	0.4943
10	83.08%	0.8210	0.8321	0.8210	0.4522

#### Configuration B: Integrated Gaussian, DFT, JPEG compression, and randomized smoothing

The client-wise performance metrics shown in table 12 vary across different clients. Clients 1, 2, 4, 5, 7, 8, 9, and 10 performed well with high training and testing accuracy, all exceeding 94% accuracy on both training and testing sets. These clients are classified as “Normal,” indicating no adversarial interference. However, Clients 3 and 6 experienced significant performance degradation, with testing accuracies of 9.82% and 9.80%, respectively, despite having relatively high training accuracies of 63.93% and 62.47%.

**Table 12:** Phase 3 Configuration B - Client-wise Performance Metrics for Integrated Gaussian, DFT, JPEG Compression, and Randomized Smoothing.

Client	Training accuracy	Testing accuracy	Attack status
Client 1	94.47%	97.34%	Normal
Client 2	95.25%	96.55%	Normal
Client 3	63.93%	9.82%	Attacked
Client 4	95.05%	96.36%	Normal
Client 5	94.75%	96.35%	Normal
Client 6	62.47%	9.80%	Attacked
Client 7	95.18%	97.01%	Normal
Client 8	95.07%	97.28%	Normal
Client 9	95.10%	96.98%	Normal
Client 10	94.57%	96.05%	Normal

**Table 13:** Phase 3 Configuration B - Training Progression Over Epochs.

Epoch	Accuracy	Precision	Recall	F1 score	Loss rate
1	75.39%	0.7422	0.7422	0.7422	1.9936
3	91.31%	0.9125	0.9125	0.9125	0.2750
5	94.54%	0.9453	0.9453	0.9453	0.1915
7	96.25%	0.9624	0.9624	0.9624	0.1227
10	97.34%	0.9734	0.9734	0.9734	0.0857

**Fig. 7:**
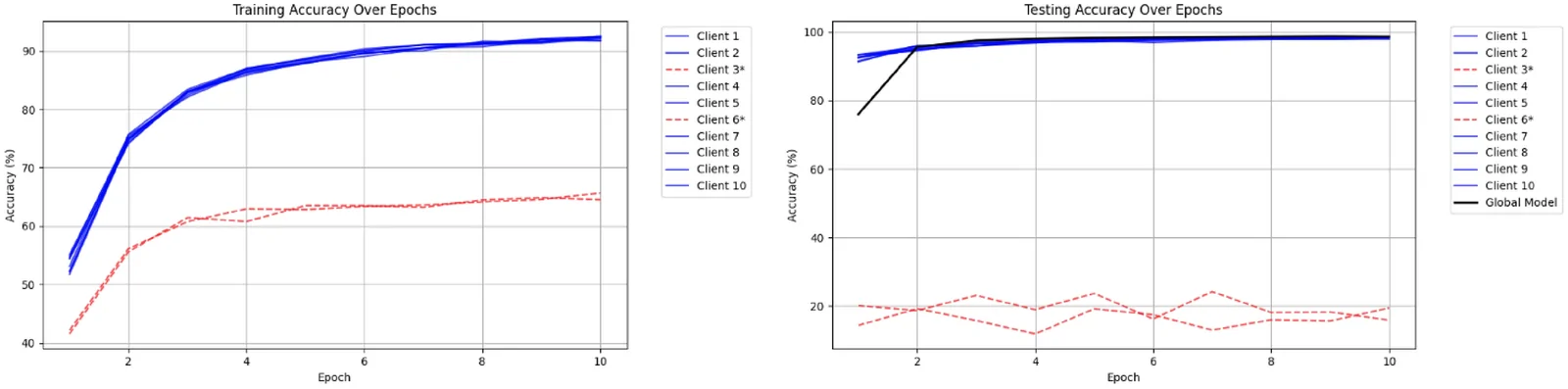
Phase 3 Configuration B - Training and Testing Accuracy of Client and Global Models for Integrated Gaussian, DFT, JPEG Compression, and Randomized Smoothing.

**Fig. 8:**
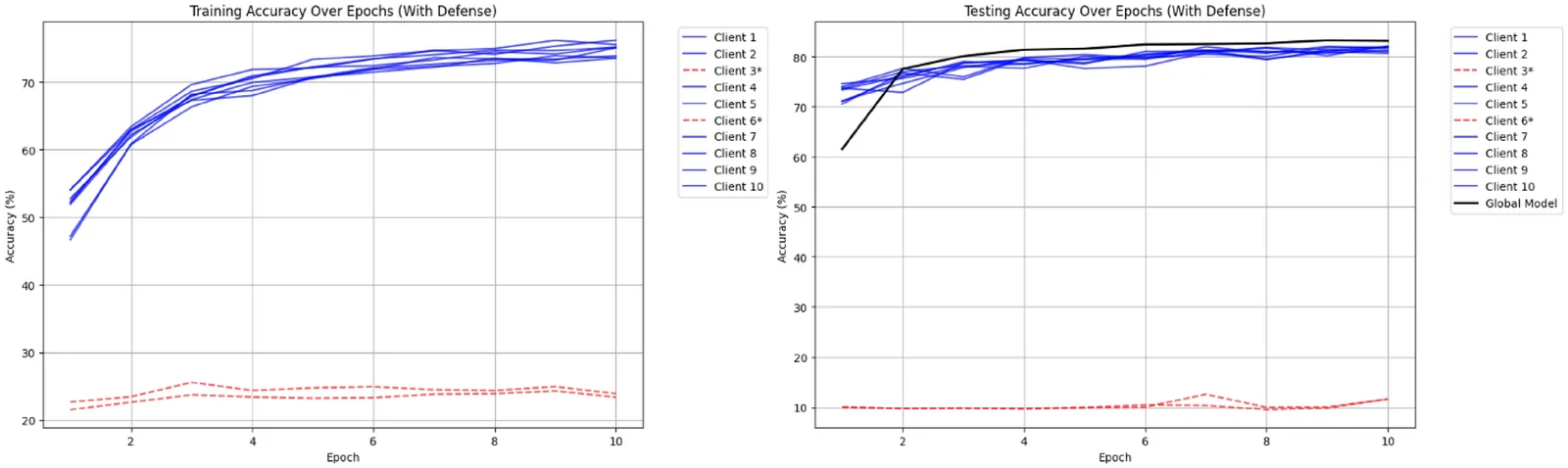
Phase 3 Configuration C - Training and Testing Accuracy of Client and Global Models for Integrated Gaussian, DFT, Differential Privacy, and Adversarial Logit Pairing.

**Fig. 9:**
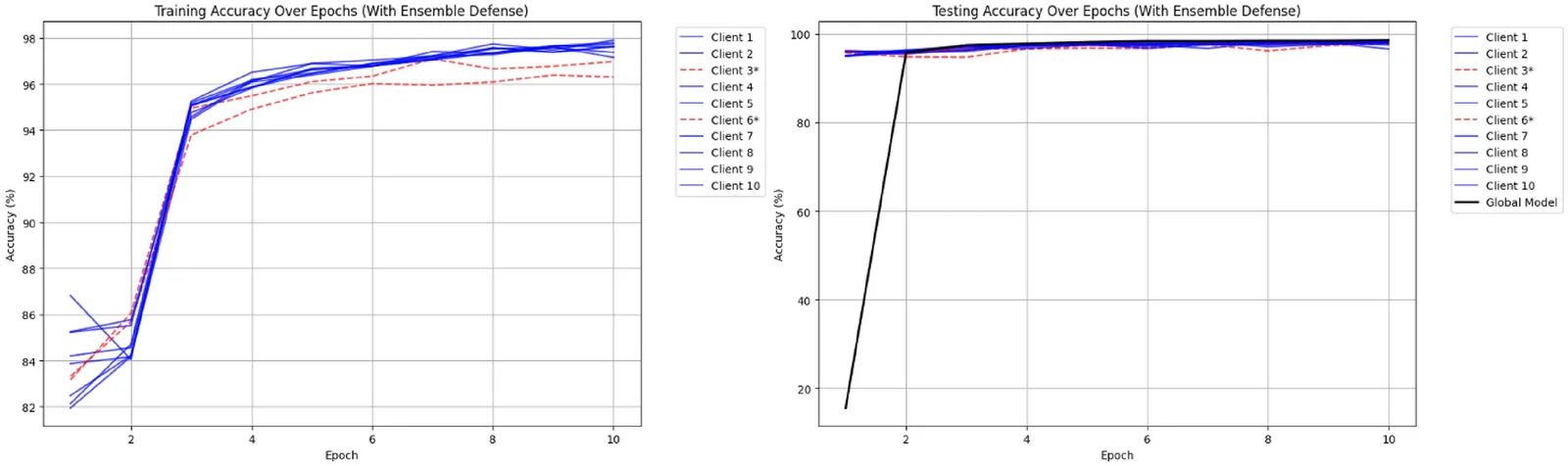
Phase 3 Configuration D - Training and Testing Accuracy of Client and Global Models.

The training progression metrics, as shown in Table 13, demonstrate a steady improvement in model performance over the epochs. Starting from epoch 1, the accuracy is 75.39%, with precision, recall, and F1 score all initially at 0.7422, indicating room for improvement. By epoch 3, the accuracy improves significantly to 91.31%, with the precision, recall, and F1 score rising to 0.9125, signaling notable progress. By epoch 5, the model achieves 94.54% accuracy, and precision, recall, and F1 score reach 0.9453, continuing this upward trend. The most significant improvement occurs by epoch 10, where the model achieves a high accuracy of 97.34%, with precision, recall, and F1 score all at 0.9734, showing excellent performance. Concurrently, the loss rate decreases consistently from 1.9936 at epoch 1 to 0.0857 at epoch 10, demonstrating that the model is effectively learning and minimizing errors over time (Fig.7, 8 and 9).

The model achieved a test accuracy of 97.34% and a training accuracy of 96.25%, with its best performance recorded at epoch 10. The convergence time was approximately 35 minutes on a CPU, demonstrating efficient training and optimization.

The enhanced defense mechanisms in Phase 3.b exhibited remarkable improvements over Phase 2C. Global accuracy showed a **+71.40%** improvement, while the loss rate was reduced by**-2.2516**. Normal client testing accuracy experienced an approximate *∼***52%** improvement, reflecting enhanced model reliability. Furthermore, a significant increase in model stability and attack resistance was observed, alongside substantial enhancements in attacked client training accuracy, reinforcing the effectiveness of the implemented defense strategies.

#### Configuration C: Integrated Gaussian, DFT, differential privacy, and adversarial logit pairing

The advanced defense mechanisms demonstrate significant improvements over previous phases, as shown in tables 14 and 15. Global accuracy increased by **+64.04%**, while the loss rate was reduced by**-1.8514**, indicating more efficient learning. Normal client testing accuracy improved by approximately *∼***47%**, highlighting the effectiveness of the enhancements. Additionally, the model exhibited enhanced stability while preserving privacy and showed strong resistance against adversarial attacks, reinforcing its robustness in secure environments.

**Table 14:** Phase 3 Configuration C - Client-wise Performance Metrics for Integrated Gaussian, DFT, Differential Privacy, and Adversarial Logit Pairing.

Client	Training accuracy	Testing accuracy	Attack status
Client 1	86.77%	88.29%	Normal
Client 2	87.12%	88.67%	Normal
Client 3	9.45%	10.32%	Attacked
Client 4	86.12%	89.46%	Normal
Client 5	86.13%	92.31%	Normal
Client 6	9.85%	10.32%	Attacked
Client 7	86.22%	88.53%	Normal
Client 8	86.27%	88.19%	Normal
Client 9	85.83%	89.76%	Normal
Client 10	86.22%	89.57%	Normal

**Table 15:** Phase 3 Configuration C - Training Progression Over Epochs.

Epoch	Accuracy	Precision	Recall	F1 Score	Loss Rate
1	58.97%	0.5360	0.5360	0.5360	2.0783
3	83.33%	0.8025	0.8025	0.8025	0.7013
5	86.19%	0.8362	0.8362	0.8362	0.5442
7	86.51%	0.8385	0.8385	0.8385	0.4832
10	89.98%	0.8971	0.8971	0.8971	0.4859

**Table 16:** Phase 3 Configuration D - Client-wise Performance Metrics for Integrated Gaussian, DFT, Differential Privacy, and Adversarial Logit Pairing.

Client	Training accuracy	Testing accuracy	Attack status
Client 1	92.97%	97.70%	Normal
Client 2	92.68%	97.38%	Normal
Client 3	89.85%	9.82%	Attacked
Client 4	92.83%	97.09%	Normal
Client 5	92.87%	97.10%	Normal
Client 6	89.30%	9.80%	Attacked
Client 7	92.68%	96.45%	Normal
Client 8	92.42%	96.48%	Normal
Client 9	92.68%	97.73%	Normal
Client 10	92.83%	97.31%	Normal

**Table 17:** Phase 3 Configuration D - Training Progression Over Epochs for Integrated Gaussian, DFT, Differential Privacy, and Adversarial Logit Pairing.

Epoch	Accuracy	Precision	Recall	F1 Score	Loss Rate
1	75.39%	0.7422	0.7422	0.7422	1.9936
3	91.31%	0.9125	0.9125	0.9125	0.2750
5	97.34%	0.9734	0.9734	0.9734	0.0857
7	97.88%	0.9788	0.9788	0.9788	0.0649
10	98.21%	0.9821	0.9821	0.9821	0.0535

#### Configuration D: Integrated Gaussian, DFT, ensemble defenses, and adversarial training

Tables 16 and 17 presents the metrics for this phase. The ensemble defense approach demonstrates exceptional improvements, achieving a **+72.27%** increase in global accuracy and a significant**-2.2838** reduction in the loss rate. Normal client testing accuracy improved by approximately *∼***53%**, further validating the effectiveness of the defense mechanisms. Additionally, a remarkable increase in attacked client training accuracy was observed, along with superior model stability and convergence, reinforcing the robustness and efficiency of the implemented approach.

### Performance comparison of defense methods

The experimental results across different defense configurations revealed notable variations in performance and resource utilization. Table 18 presents a comprehensive comparison of the defense mechanisms across key metrics:

**Table 18:** Performance Comparison of Defense Mechanisms.

Metric	Phase 3 config A	Phase 3 config B	Phase 3 config C	Phase 3 config D
Model Accuracy (%)	84.64	88.60	89.70	98.21
Attack Resistance (%)	75.39	83.33	86.19	97.88
Recovery Rate (%)	82.42	84.65	88.39	96.25
Processing Time (ms)	46.36	62.53	73.87	249.86
Memory Usage (MB)	128	256	384	512

While the proposed defenses demonstrate substantial robustness gains, these improvements come with varying computational costs, as reflected in the processing time and memory usage reported in Table 18. The processing time reported corresponds to the average additional local computational overhead incurred at each client during a federated training round, excluding communication latency. In particular, configurations incorporating ensembles or multi-stage filtering introduce higher latency and resource consumption, which may limit practical deployment in constrained FL environments. This highlights an important trade-off between defense strength and system feasibility, motivating future work toward lighter-weight cascade variants.

### Real-world implementation considerations

The deployment of these defense mechanisms in real-world scenarios requires careful consideration of practical factors. The experimental results from Integrated Gaussian, DFT, Ensemble Defenses, and Adversarial Training showed that while achieving 98.21% accuracy, the system required significant computational resources, with processing times of 249.86 ms per batch. In production environments, this overhead must be carefully weighed against security requirements.

A detailed analysis of system requirements across different deployment scales revealed varying resource needs shown in Table 19.

**Table 19:** Implementation Requirements by Deployment Scale.

Scale	Clients	Memory/ Client	Processing Power	Network Bandwidth
Small (*≤*10)	5–10	512 MB	4 CPU Cores	100 Mbps
Medium (11–50)	11–50	1 GB	8 CPU Cores	500 Mbps
Large (51–100)	51–100	2 GB	16 CPU Cores	1 Gbps
Enterprise (>100)	>100	4 GB	32 CPU Cores	10 Gbps

## Conclusions and future work

The results demonstrate that applying multi-layered defense mechanisms significantly enhances model robustness under adversarial attack conditions. Among the implemented strategies, Integrated Gaussian, DFT, Ensemble Defenses, and Adversarial Training method achieve the highest accuracy of 98.21%. This highlights the effectiveness of ensemble defenses, where techniques such as Gaussian Filtering, Fourier Transform, and Randomized Smoothing work in a complementary manner. Based on our findings, several promising research directions emerge for further investigation, for example, the use of adaptive defense mechanisms or low defense mechanisms that employ low computational overheads. Future work will also focus on evaluating the cascade defense under adaptive and black-box attacks, to further assess robustness beyond gradient-based settings.

## Data Availability

This study utilized publicly available datasets. The MNIST dataset can be accessed at https://docs.ultralytics.com/datasets/ classify/mnist/.
